# Poly(3-Hydroxybutyrate)-Multiwalled Carbon Nanotubes Electrospun Scaffolds Modified with Curcumin

**DOI:** 10.3390/polym12112588

**Published:** 2020-11-04

**Authors:** Nader Tanideh, Negar Azarpira, Najmeh Sarafraz, Shahrokh Zare, Aida Rowshanghiyas, Nima Farshidfar, Aida Iraji, Moein Zarei, Miroslawa El Fray

**Affiliations:** 1 Stem Cell Technology Research Center, Shiraz University of Medical Sciences, Shiraz 71348-14336, Iran; tanidehn@gmail.com (N.T.); zare.shahrokh@gmail.com (S.Z.); 2 Pharmacology Department, Shiraz University of Medical Sciences, Shiraz 71348-14336, Iran; 3 Transplant Research Center, Shiraz University of Medical Sciences, Shiraz 71348-14336, Iran; negarazarpira@gmail.com; 4 Department of Periodontics, School of Dentistry, Shiraz University of Medical Sciences, Shiraz 71348-14336, Iran; najmehsarafraz@yahoo.com; 5 Department of Medical Biotechnology, Tehran Medical Science, Islamic Azad University, Tehran 19395-1495, Iran; a.rowshanghias@gmail.com; 6 Student Research Committee, Shiraz University of Medical Sciences, Shiraz 71348-14336, Iran; n.farshidfar@icloud.com; 7 Medicinal and Natural Products Chemistry Research Center, Shiraz University of Medical Sciences, Shiraz 71348-14336, Iran; aida.iraji@gmail.com; 8 Department of Polymer and Biomaterials Science, Faculty of Chemical Technology and Engineering, West Pomeranian University of Technology, Szczecin, Al. Piastow 45, 71-311 Szczecin, Poland

**Keywords:** P3HB, curcumin, carbon nanotubes, tissue engineering, scaffolds, electrospinning

## Abstract

Appropriate selection of suitable materials and methods is essential for scaffolds fabrication in tissue engineering. The major challenge is to mimic the structure and functions of the extracellular matrix (ECM) of the native tissues. In this study, an optimized 3D structure containing poly(3-hydroxybutyrate) (P3HB), multiwalled carbon nanotubes (MCNTs) and curcumin (CUR) was created by electrospinning a novel biomimetic scaffold. CUR, a natural anti-inflammatory compound, has been selected as a bioactive component to increase the biocompatibility and reduce the potential inflammatory reaction of electrospun scaffolds. The presence of CUR in electrospun scaffolds was confirmed by ^1^H NMR and Fourier-transform infrared spectroscopy (FTIR). Scanning electron microscopy (SEM) revealed highly interconnected porosity of the obtained 3D structures. Addition of up to 20 wt% CUR has enhanced mechanical properties of the scaffolds. CUR has also promoted in vitro bioactivity and hydrolytic degradation of the electrospun nanofibers. The developed P3HB-MCNT composite scaffolds containing 20 wt% of CUR revealed excellent in vitro cytocompatibility using mesenchymal stem cells and in vivo biocompatibility in rat animal model study. Importantly, the reduced inflammatory reaction in the rat model after 8 weeks of implantation has also been observed for scaffolds modified with CUR. Overall, newly developed P3HB-MCNTs-CUR electrospun scaffolds have demonstrated their high potential for tissue engineering applications.

## 1. Introduction

Scaffolding, based on biodegradable materials (mostly polymers) are playing a central role in tissue engineering along with living cells and bioactive factors to modulate new tissue functions [1,2]. The most important features of scaffolding structures are sufficient mechanical properties, interconnected porosity, biocompatibility and biodegradability [3,4]. To fulfill these requirements, different fabrication methods, such as solvent casting, freeze drying, gas foaming, electrospinning and bioprinting have been tried to create various structures mimicking native tissues of the human body [5]. Since the morphology of the extracellular matrix (ECM) of the human body resembles fibrous networks, using nanofibrous structures has a tangible effect on promoting cell attachment and migration [6]. Various techniques have been applied to create nanofibers, including self-assembly, template synthesis and thermally-induced phase separation. However, electrospinning has attracted the most attention, owing to the low cost and the ability to create different nanofibrous structures. With rapid development of the electrospinning technique, different fibrous structures with unique properties, such as co-axial, tri-axial, side-by-side, and emulsion electrospinning for creating core-shell and other complicated nanostructures, can now be created by the modification of the spinneret and the type of solution [7,8,9]. Similarly, various natural and synthetic biomaterials have been extensively applied for the creation of scaffolds; the greatest success has been achieved with biodegradable biopolymers [10,11,12]. Single-fluid blending electrospinning was used according to its merits, which are facility to conduct, ease to be scaled up, and a powerful ability to tailor the components and compositions of resultant composite nanofibers [10].

Bacteria-synthesized biopolymers, polyhydroxyalkanoates (PHAs), have triggered special attention in the development of various medical devices, soft and hard tissue engineering applications and drug delivery systems [13]. Among various PHAs, poly(3-hydroxybutyrate) (P3HB), has been considered for diverse medical applications in recent decades. Biodegradability of P3HB through simple hydrolysis creates a metabolite of the human body, butyric acid [14]. In the field of tissue engineering, P3HB has also been used for producing tissue engineering scaffolds by electrospinning and the results for these studies are promising for both soft and hard tissue engineering applications [15,16]. Biocompatibility of P3HB, good solubility in organic solvents and easy fabrication of well-integrated fibers similar to ECM via electrospinning have made the selection of the P3HB biopolymer a natural choice to create 3D structures suitable for making tissue engineering scaffolds [15,17]. However, P3HB scaffolds are not free from problems, as their lack of mechanical strength is a significant limitation for hard tissue engineering applications [18]. Thus, different biomaterials, such as bioglass, hydroxyapatite, chitosan, silk, keratin and carbon nanotubes were tried to improve mechanical and also biological properties of P3HB, and as a result, the properties of modified P3HB were significantly improved [1,19,20,21,22,23]. Especially promising for tissue engineering are nanofibers used as reinforcements [17]. Functionalized multiwalled carbon nanotubes (MCNTs), with their unique physical and biological properties such as extraordinary mechanical strength and modulus and excellent compatibility in contact with blood and tissues, have been one of the best options to improve the mechanical and biological properties in different composite structures used in medical devices [24,25]. According to the reports on their biocompatibility, although MCNTs without functionalization have shown some negative effects in contact with cells and animals, functionalization of MCNTs make them inert or enhance the biocompatibility of the structures [26]. Heterogeneous functionalization or surface chemistry of MCNTs, including the formation of carboxyl groups at the sidewalls and also at the end of the tubes, was shown to increase both in vitro and in vivo biocompatibility [26,27,28]. Therefore, MCNTs have already been reported to improve not only mechanical properties, but also the biological response in the presence of different polymers, including P3HB [21,28,29].

P3HB-MCNTs nanocomposite scaffolds were already studied in vitro and in vivo, and the results were promising for bone tissue engineering [21,23,27,28]. The results of those studies indicated, however, some inflammatory reaction after 6 and 8 weeks of subcutaneous implantation, albeit this inflammation was not an acute chronic one. Therefore, there is a pressing need to find a suitable material to decrease or eliminate such inflammatory reactions. A promising candidate with good anti-inflammatory and anti-oxidant properties is curcumin (CUR), a natural ingredient selected for this study [30,31]. CUR, a natural component from curcuma longa plants and a member of the ginger family, has many biological advantages and has been chosen for tissue engineering and targeted drug delivery systems in recent years [32,33]. Due to the anti-oxidative, anti-inflammatory and anti-tumoral activity of CUR, this natural active material has been selected for reducing the inflammations in different studies [33]. According to various studies, CUR triggered anti-inflammatory, bioactivity and biocompatibility in different structures, when combined with silk, gelatin, collagen and chitosan [34,35,36,37]. CUR has also been studied in combination with different polymers to produce electrospun scaffolds. Poly(ε-caprolactone) (PCL) electrospun fibers loaded with CUR were shown to improve the biocompatibility, cell viability and antibacterial activity of scaffolds [38,39,40]. The same results were reported for poly(lactic acid) (PLA), polyvinyl alcohol (PVA), polyurethanes (PU) and poly (lactic-co-glycolic acid) (PLGA) copolymer electrospun scaffolds loaded with CUR [41,42,43,44]. The osteoblast differentiation of mesenchymal stem cells (MSC) was encouraged by using a specific concentration of CUR, thus indicating a high potential for bone regeneration, especially at a low concentration [45].

With this in mind, we combined P3HB-MCNTs for the first time with CUR to reduce the possibility of inflammation of electrospun nanofibrous scaffolds in their use as potential structures for bone tissue engineering applications. It has recently been reported that the addition of only 0.5 wt% of MCNTs was optimal and significantly increased the tensile strength of P3HB nanofibers [21]. Therefore, P3HB and 0.5 wt% of MCNTs nanocomposite scaffolds were selected to be loaded with CUR. Different concentrations of CUR were tested to determine the optimum composition in terms of favorable physical, mechanical and biological properties.

## 2. Materials and Methods

### 2.1. Materials

P3HB powder with the formulation of –[COCH_2_CH(CH_3_)O]**_n_**- and CAS Number: 29435-48-1, CUR (CUR)(CAS Number: 458-37-7), chloroform (formula: CHCl_3_, CAS Number: 67-66-3), dimethylformamide (DMF) (formula: HCON(CH_3_)_2_, CAS Number: 68-12-2), dimethyl sulfoxide (DMSO) (Formula: (CH_3_)_2_SO, CAS Number: 67-68-5), DMEM F12, fetal bovine serum (FBS) (MDL Number: MFCD00132239) and MTT formazan (CAS Number: 57360-69-7) were purchased from Sigma-Aldrich-Merck (St. Louis, MO, USA). MCNTs (5‒25 nm in diameter, 0.5‒2 μm in length, and >95wt%. of purity) functionalized with carboxylic groups (–COOH) were obtained from US Research Nanomaterials (Houston, TX, USA).

### 2.2. Electrospinning of Fibrous Scaffolds

A mixture of chloroform and DMF in the ratio of 7:3 *v/v* was used to prepare the P3HB solution at a concentration of 6 *w/v* %. Then, 0.5 wt% of MWNTs was added to the solution and ultra-sonicated for 1 h at a temperature of 60 °C and amplitude of 70%. P3HB-MCNT solutions were then used to prepare the final solutions for electrospinning with different amounts of CUR, namely 10%, 20% and 30 wt%. After achieving transparent and viscous liquids, the solutions were loaded into a 5 mL syringe and were inserted into the electrospinning device. The electrospinning device was used in a horizontal configuration with aluminum foil as the collector, a syringe pump and an adjustable 0–35 kV DC power supply (FNM Co., Tehran, Iran). Electrospinning parameters like distance between the tip of the needle and collector, applied voltage and flow rate were optimized.

### 2.3. Chemical and Structural Characterization

A Bruker Ascend 300 spectrometer (300 MHz) was used to record spectra of ^1^H NMR (128 scans, 1 s relaxation delay). The samples were dissolved in CDCl_3_ (~40 mg/mL) and tetramethylsilane (TMS) was employed as an internal reference for the reported chemical shifts. Fourier-transform infrared spectroscopy (FTIR) (FTIR-JASCO 6300, JASCO Deutschland GmbH, Pfungstadt, Germany) spectra were collected for P3HB, MCNTs and CUR powders and attenuated total reflection (ATR) spectra were obtained for the electrospun composite scaffolds. The FTIR and ATR spectra were recorded from 400 to 4000 cm^−1^ at a resolution of 0.5 cm^−1^ with 32 scans. Scanning electron microscopy (SEM) (VEGA TESCAN-LMU, Brno, Czech Republic) was used to evaluate the surface morphology of the fibrous structures. SEM micrographs were analyzed with Image J and Matlab software to evaluate the mean fiber diameters and the percentage of porosity of the structures. For evaluating the mean fiber diameter of each group and for calculating the standard deviation (SD), 50 fiber diameters from SEM micrographs were measured by Image J software. The porosity of fibrous structures was evaluated for 5 different SEM images with the same scale for each group, using Matlab software [46].

### 2.4. Mechanical Properties

Mechanical properties of fibrous structures were assessed with tensile tests using a universal testing machine (Santam, Tehran, Iran) according to the ISO 1798. The samples were prepared in 10 mm × 50 mm (width × length) squares and 250 µm thickness and the strain rate was 1 cm/min. Measurements were carried out for each group of scaffolds and results were averaged from 5 specimens. The tensile strengths of the scaffolds were calculated from the stress–strain curves and the average value with standard deviation has been provided.

### 2.5. Hydrolytic Degradation

The degradation of scaffolds was measured by immersing them into the phosphate buffer saline (PBS) at pH = 7.4 and kept at 37 °C for 4 weeks. Scaffolds were weighted at each time point to calculate the mass loss, expressing the percentage of degradation. The mass loss, WL was calculated for each group according to the following equation:

WL = (Wf − Wr)/Wf × 100(1)

where Wf represents the dry weight of the scaffolds before degradation and Wr is the remaining weight after each time point. The WL was calculated as the average from 5 samples.

### 2.6. In Vitro Bioactivity

In vitro bioactivity of the scaffolds was carried out by immersing samples in simulated body fluid (SBF). Briefly, the scaffolds were cut into 10 mm × 10 mm squares and were immersed into the falcons filled with 40 mL SBF. The falcons were then placed into an incubator for four weeks, in static conditions at 37 °C. The bioactivity demonstrated by hydroxyapatite crystals precipitation on the surface of the scaffolds was examined with SEM imaging.

### 2.7. Stem Cells Isolation and Identification

The synovial mesenchymal stem cells (SMSCs) were extracted from the synovial membranes of rats according to the previous report [22]. Briefly, the membranes were digested by using 1 mg/mL collagenase type D (CD) solution for 2 h and enzyme activity was stopped by adding DMEM cell culture medium. The solution was then filtered and centrifuged to obtain the extracted cells. DMEM cell culture medium containing penicillin/streptomycin 1%, and FBS 10% was added into the cell culture flasks; the extracted cells were moved to these flasks and were kept in the incubator at 37 °C with 95% humidity and 5% CO_2_.

In order to identify stem cells, three different methods were applied. The first one was carried out after the 3rd passage of extracted SMSCs, by using the expression of surface markers by flow cytometry to evaluate the positive surface biomarkers expression of CD44 and CD90 for mesenchymal stem cells and negative surface biomarkers expression for CD34 and CD45 for hematopoietic stem cells. The osteogenic differentiation potential is one of characteristic features of mesenchymal stem cells. In the second method, the extracted SMSCs osteogenic differentiation ability was evaluated by culturing them in low glucose DMEM supplemented with 0.05-mM ascorbate-2-phosphate, 10-mM β-glycerophosphate, 100-nM dexamethasone, 15% FBS, and 1% antibiotic/antimycotic (osteogenic medium). Microscopic pictures (Nikon, Eclipse TS10, Nikon, Tokyo, Japan) were taken after 21 days of culture. In the third method, the morphology of the extracted SMSCs after the first and third days of extraction was evaluated. A Nikon Eclipse TS10 was used to monitor the attachment and form of extracted SMSCs.

### 2.8. In Vitro Biocompatibility

The scaffolds were cut into discs with 5 mm diameter and placed into packaging sleeves for plasma autoclave sterilization. SEM images and MTT assay were applied for evaluating the cellular behavior of extracted SMSCs in direct contact with the scaffolds. 96-well cell culture plates were used for this study to insert scaffolds of 5 mm in diameter fitting into a single well. Then, the SMSCs with a concentration of 105 per well were seeded onto the scaffolds and kept in the incubator for 10 days. The culture medium used for this study was DMEM F12 containing 10% FBS and 1% penicillin/streptomycin which was replaced by a fresh medium every day. After the first and the tenth day of culture, the cells seeded on the scaffolds were fixed with 2.5% glutaraldehyde to evaluate the SMSCs attachment to the scaffolds. In order to evaluate the cell viability, the MTT solution with cell medium was mixed and after 3 h, DMSO was added and centrifuged for 10 min. The optical density at 570 nm was acquired for each group using an ELISA plate reader (Polar Star Omega, BMG Labtech, Ortenberg, Germany). 10 scaffolds were used for each group.

### 2.9. In Vivo Biocompatibility

The selected animals for evaluating the biocompatibility of the scaffolds were 8 to 10 weeks old male Sprague-Dawley rats with a weight range between 220 to 240 g; the rats were obtained from the animal laboratory center, Shiraz University of Medical Sciences, Shiraz, Iran. The animal study, including anesthesia, surgery, animal care and euthanasia was performed according Code of Practice for the Housing and Care of Animals Used in Scientific Procedures, Act 1986 and under ethical consideration of general principles and rules for animal studies by Animal Care and Use Committee, Shiraz University of Medical Sciences, Shiraz, Iran (Approval No: IR.SUM. 98-01-67-20175). The dorsal section of the rats was chosen as the implantation site. An aseptic surgery method was applied for implanting the scaffolds under the skin. The rats were anesthetized and prepared for surgery by using a 2:1 ratio of 10% ketamine and 2% xylazine, respectively. A 15 mm incision was made in the site, the scaffold was implanted, and the skin was sutured. For protecting the eyes of the rat from being dry, ophthalmic liquid gel was used during the surgery. After that, the rats were kept under standard condition in the animal laboratory for 8 weeks. The in vivo biocompatibility of the scaffolds was carried out by evaluating the reaction of the body and host surrounding tissues to scaffolds after 8 weeks in time intervals of 1, 4 and 8 weeks, respectively. In total, 54 samples were used (18 samples for each time period; 6 samples for each group of scaffolds). The scaffolds were first cut in the dimension of 15 mm × 15 mm and then sterilized under a plasma autoclave. They were then implanted in the dorsal section of the rats. The scaffolds and tissues from that section were harvested after 1, 4 and 8 weeks and then fixed in 10% formalin. A histological study, including evaluation of inflammation, cell infiltration and vascularization, was performed by a blind pathologist with hematoxylin–eosin (H&E) staining. An optical microscope (BX53F Olympus, Tokyo, Japan) was used for morphological analysis.

### 2.10. Statistical Analysis

All the examinations were done in at least 3 repetitions. Data was analyzed using one-way analysis of variance (ANOVA) and the Mann-Whitney U test by SPSS software (version 21) and the significance was considered at *p* ≤ 0.05.

## 3. Results and Discussion

### 3.1. Chemical Structure

**Figure 1:**
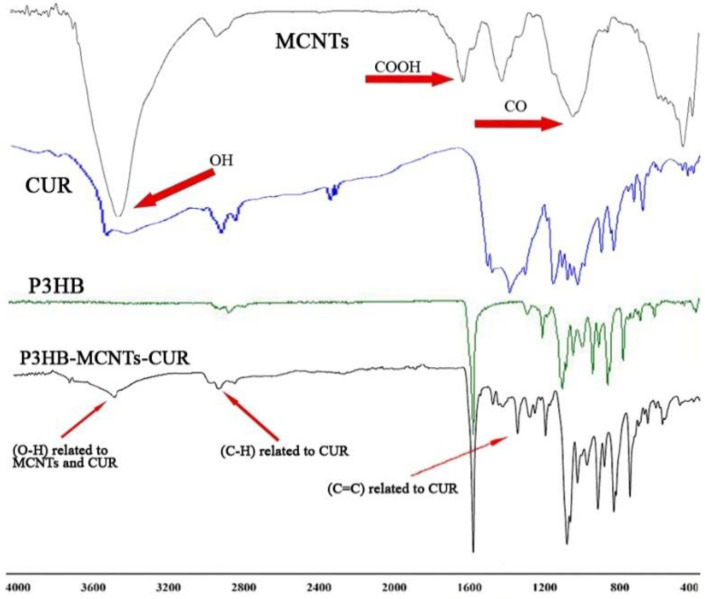
Infrared spectra of the neat materials and P3HB-MCNTs scaffold loaded with 20 wt% of curcumin (CUR). **Abbreviations:** P3HB: poly(3-hydroxybutyrate; MCNTs: multiwalled carbon nanotubes.

P3HB-MCNT composites were loaded with different contents of CUR and their chemical structure was verified with NMR and IR spectroscopy. FTIR spectra were acquired for P3HB, MCNT and CUR powders to evaluate the chemical structure of the neat materials. The ATR module was used to examine the chemical structure of composite scaffolds containing 20 wt% CUR. Figure 1 shows the spectra of the neat materials and the composite scaffolds, which confirms that both MCNTs and CUR are present in the scaffold. Incorporation of MCNTs functionalized with -COOH groups was confirmed by two absorption bands at 1427 and 1630 cm^−1^, which are ascribed to vibration modes of C−C and two bands at 870 and 1150 cm^−1^ referring to C−O groups [46]. The hydroxyl groups of the MCNTs were identified by broad bands at 2190 and 3430 cm^−1^. The chemical structure of CUR was confirmed by olefinic vibration of C-H, the stretching vibration of C=C and the carbonyl group C=O bonds identified at around 1152, 1508 and 1625 cm^−1^. In addition, phenolic O-H and aromatic C−H vibrations appeared at 3504 and 2922 cm^−1^, respectively. For composite scaffold, characteristic bands related to the C=C and C=O bonds of CUR were found at around 1500–1600 cm^−1^ and another peak related to the C−H vibration of CUR was found at 2930 cm^−1^. Additionally, a broad band at 3500 cm^−1^, related to both MCNTs and CUR was also found.

**Figure 2:**
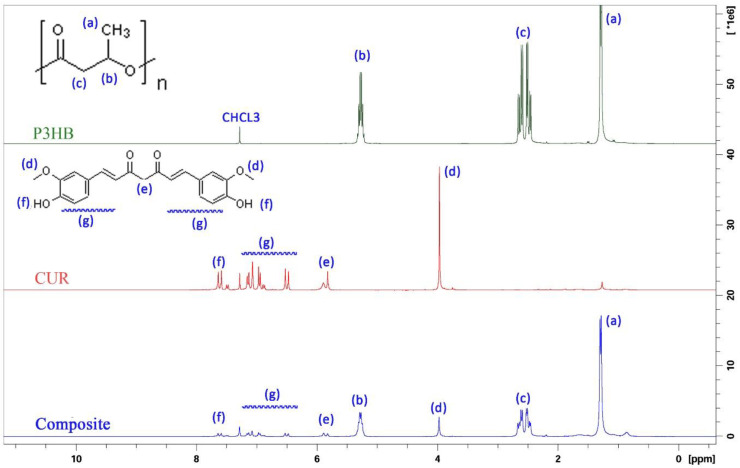
^1^H NMR spectra of P3HB, CUR and the P3HB-MWCNT-CUR mixture.

^1^H NMR has been performed to identify chemical shifts of the neat P3HB polymer and bioactive compound, CUR. Moreover, spectral characterization has also been performed for the P3HB-MWCNT-CUR mixture. All NMR spectra are grouped in Figure 2. For P3HB, the doublet located at δ = 1.28 ppm is ascribed to the methyl (a) group. Distinct signals detected at δ = 2.45–2.66 ppm, and at δ = 5.27 ppm belong to the −CH_2_ multiplet (b) and the −CH sextet (c), respectively. Analysis of CUR spectra revealed a signal at δ = 3.97 ppm corresponding to the methoxy groups (d), while signals at δ = 5.81 ppm and δ = 5.82 ppm belong to the −CH_2_− bridge (e). The appearance of a proton signal at δ = 6.7–7.5 ppm indicates the presence of alkene and aromatic moieties (g). Finally, the P3HB-MCNTs-CUR mixture reveals the change in chemical shifts of the aromatic part of curcumin and methylene bridge protons (b, d) which were shifted downfield. The chemical shifts of components and the composite scaffold are presented in Figure 2. It has already been shown that carboxyl groups (−COOH) on the MCNTs surface and end walls may form covalent bonds with hydroxyl groups of P3HB and with CUR [24]. However, we could not undoubtedly confirm such strong interactions since simple mixing has been applied for our system preparation.

Table 1 summarizes all chemical shifts with ascribed chemical groups and the integral value.

### 3.2. Characterization of Fibrous Scaffolds

**Figure 3:**
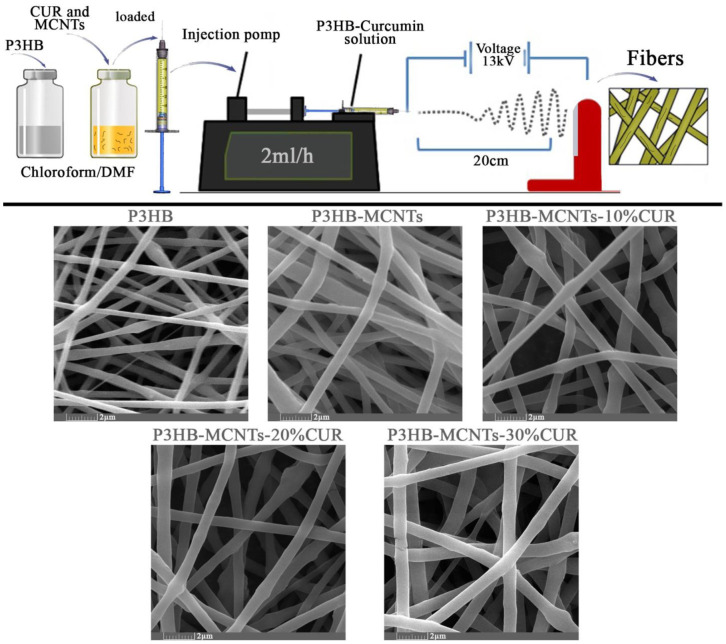
Upper panel: schematic representation of the scaffolds preparation setup with optimum parameters; lower panel—scanning electron microscopy (SEM) micrographs of fabricated scaffolds using neat P3HB, P3HB-MCNTs nanocomposite, and P3HB-MCNTs loaded with 10, 20 and 30 wt% of CUR, respectively.

The morphology of the scaffold structure, including the porosity and interconnectivity of pores, is an important feature for tissue engineering, to have an effect on cell function. Electrospinning is known as an excellent way to produce fibrous scaffolds. Polymer solution viscosity is one of the most important factors in the electrospinning method; it contributes to the final shape and diameter of the nanofibers. In this study, 5 different solutions with different concentrations were prepared: group 1 and 2 were neat P3HB and P3HB/0.5 wt% MCNTs respectively, and groups 3, 4 and 5 were P3HB/0.5 wt% MCNTs with 10%, 20% and 30 wt% of CUR, respectively. All solutions were well dispersed and transparent. In order to obtain uniform fibers, the parameters of the electrospinning process were varied. Figure 3 shows SEM micrographs with nanofiber morphology prepared at optimum parameters, namely with an applied voltage of 13 kV, the distance between the needle and the collector of 20 cm and the flow rate of 2 mL per hour.

**Figure 4:**
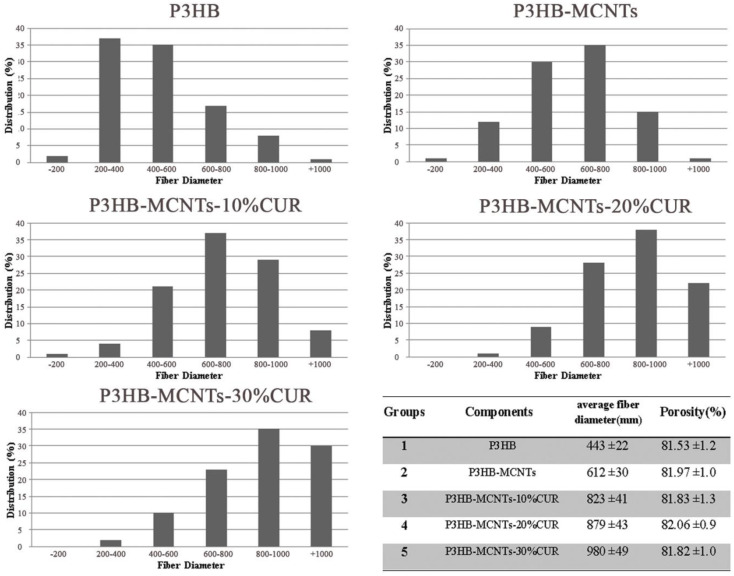
Histograms of fiber diameters and a summary of the average fiber diameter and porosity.

As can be seen from Figure 3, the resulting fibers are smooth and create a porous interconnected scaffold structure, even for the sample with a high load of 30% CUR. The orientations of the fibers were random and the size of the fibers changed with material composition. In Figure 4, the fiber diameter distribution in different groups is shown, as well as the mean fiber diameter with standard deviation (SD) and the mean porosity of each group. The mean fiber diameters were between 350 nm (for neat P3HB) to 950 nm for P3HB-MCNT composites loaded with highest amount of CUR. In most studies, it is confirmed that the additional components introduced into the composite can increase the fiber diameter and this might be due to the increased viscosity of the solution, as increasing the viscosity can result in the creation of thicker fibers [21]. We also noticed that addition of MCNTs to P3HB and additionally CUR significantly increased the fiber diameter for all concentrations. The percentage of porosity is considered the most important factor for tissue engineering applications [1]. The porosity of the scaffolds was almost uniform; the porosity was in the range of 80%, which is sufficient for cell migration and growth. Overall, the interconnected porosity and fiber diameter can provide the conditions necessary for cells to proliferate and migrate to regenerate the new tissue.

### 3.3. Mechanical Properties

**Figure 5:**
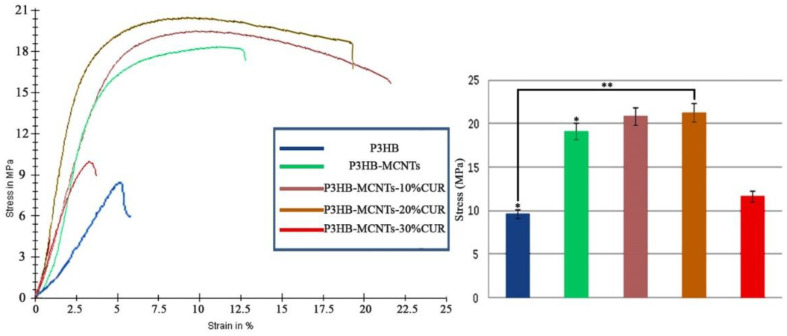
Stress–strain curves of P3HB and P3HB-MCNTs scaffolds loaded with different amounts of CUR. The right panel summarizes tensile strength values, * *p* ≤ 0.05, ** *p* ≤ 0.01.

Sufficient strength of the scaffold is another important requirement for scaffolds in tissue engineering. For different tissues, different mechanical properties, similar to the host tissue, are required. During regeneration of the new tissue, the scaffold should withstand the mechanical forces and support the cells to proliferate and migrate [47]. MCNTs are known for their outstanding mechanical properties; therefore. it is expected that they could have a positive effect on tensile strength improvement of the P3HB scaffolds. Figure 5 shows and compares the stress–strain curve of different groups of the scaffolds. It can be seen that incorporation of MCNTs into the P3HB structure significantly increased the tensile strength and modulus of scaffolds. The effect of different amounts of CUR on the mechanical properties of the scaffolds has also been studied. It was found that addition of CUR up to 20 wt% had a significant effect on increasing ultimate strength values as compared to the neat P3HB. Only at 30 wt% of CUR, decrease of tensile strength and the elongation at break has been noticed. The obtained results are comparable to other studies with CUR on electrospun structures where CUR could increase the ultimate strength up to some limit, and beyond the limit, it had negative effects [39,48]. We found that CUR loaded up to 20 wt% can be suitable in terms of maintaining good mechanical properties and is responsible for the elasticity of the scaffolds. Similar results were reported by Ranjbar-Mohammadi et al., indicating that CUR loaded in PCL electrospun structures increased their mechanical strength and elasticity [39]. In another study, an increase of tensile strength and elasticity of electrospunPLA fibers was also reported by adding CUR [42]. In our experiments, a significant decrease of mechanical properties was noticed for group 5 (scaffolds with 30% CUR) and thus it has been eliminated from further studies.

### 3.4. In Vitro Degradation

**Figure 6:**
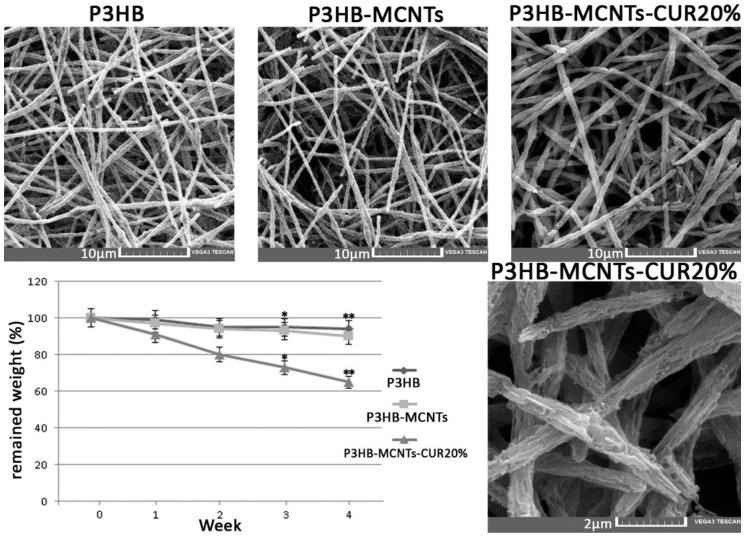
Scaffolds morphology and mass loss after 4 weeks of degradation in phosphate buffer saline (PBS), * *p* ≤ 0.05, ** *p* ≤ 0.01.

Figure 6 shows the results of in vitro hydrolytic degradation in PBS. The neat P3HB showed a 6% mass loss after 4 weeks while an increase in mass loss of 9% was noticed for P3HB-MCNTs, thus indicating more rapid degradation of composite fibers. Similar results for P3HB and P3HB-MCNTs were reported in other research work [49]. Interestingly, addition of 20% CUR increased the biodegradation rate to about 35% of mass loss after 4 weeks. SEM micrographs of the scaffolds structure clearly indicates changes on the surface of the fibers. Thus, addition of CUR can significantly increase the biodegradation rate of the scaffold and most likely can be easily released by decomposition of the scaffold to induce expected bioactivity, e.g., reduce inflammatory response as an anti-inflammatory agent.

### 3.5. Scaffolds Bioactivity in SBF

**Figure 7:**
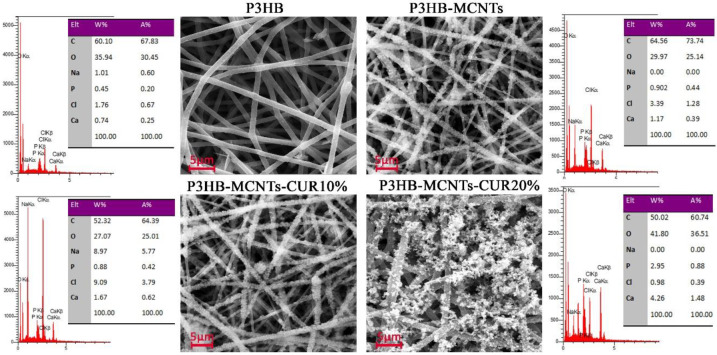
SEM micrographs of neat P3HB scaffolds and P3HB-MCNTs structures loaded with 10 and 20 wt% of CUR after 4 weeks immersing in simulated body fluid (SBF).

Bioactivity is described as the ability of a material to be accepted by the body and to bond with bone [50]. In vitro bioactivity can be assessed by immersing the material into the SBF and monitoring the surface apatite formation after a specific time period [50]. In this study, scaffolds were immersed into SBF and SEM pictures were taken after 4 weeks. As can be seen in Figure 7, some spherical crystals formed onto the surface of the fibers, which are ascribed to sedimentary hydroxyapatite (HA) particles. In order to confirm that, EDX analysis was employed during capture of the SEM micrographs; the results indicate that Ca and P atoms are detected; these are characteristic for HA. For the P3HB scaffold, only a few white points are seen on the surface of the fibers, which indicates a small amount of HA formation. More crystals can be seen after addition of MCNTs and further crystals formed after the addition of CUR. The amount of calcium was increased in the scaffold containing MCNTs, in comparison to the neat P3HB scaffold, from 0.74 to 1.17 wt%, respectively. This amount was further increased to 4.26 wt% when 20% CUR was added. The new P3HB-MCNTs nanocomposite scaffolds containing CUR show the capability to precipitate a greater amount of HA crystals onto the fibers surface thus increasing their bioactivity.

### 3.6. In Vitro Cytotoxicity

**Figure 8:**
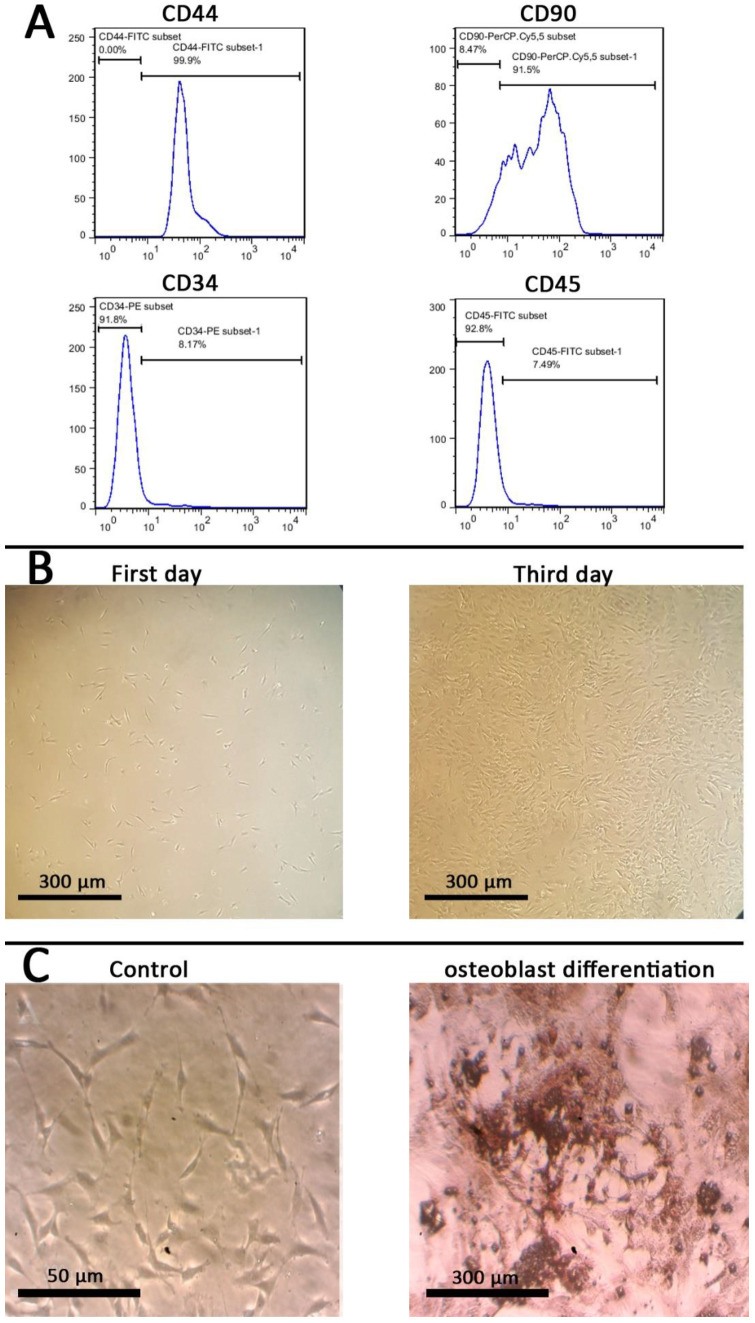
(**A**) Identification of mesenchymal stem cells (MSCs) with flow cytometry, (**B**) visualization of their morphology, and (**C**) osteoblasts differentiation.

In vitro cytotoxicity studies were performed with mesenchymal stem cells (MSCs), as they offer great promise for bone tissue engineering. Different methods were used for the identification of MSCs, including flow cytometry, morphology and evaluation of osteoblasts differentiated potential. Figure 8A shows the results of flowcytometry after the third passage. Positive results for mesenchymal stem cells markers (CD44 and CD90) indicate the presence of the stem cells, while the negative results for hematopoietic markers (CD34 and CD45) show the absence of hematopoietic stem cells. Figure 8B shows the morphologies of the extracted stem cells after the first and third days; they have a spindle-shape typical for stem cells. Figure 8C shows the biomineralization of ECM after 21 days, indicating osteoblast differentiation of the extracted stem cells.

**Figure 9:**
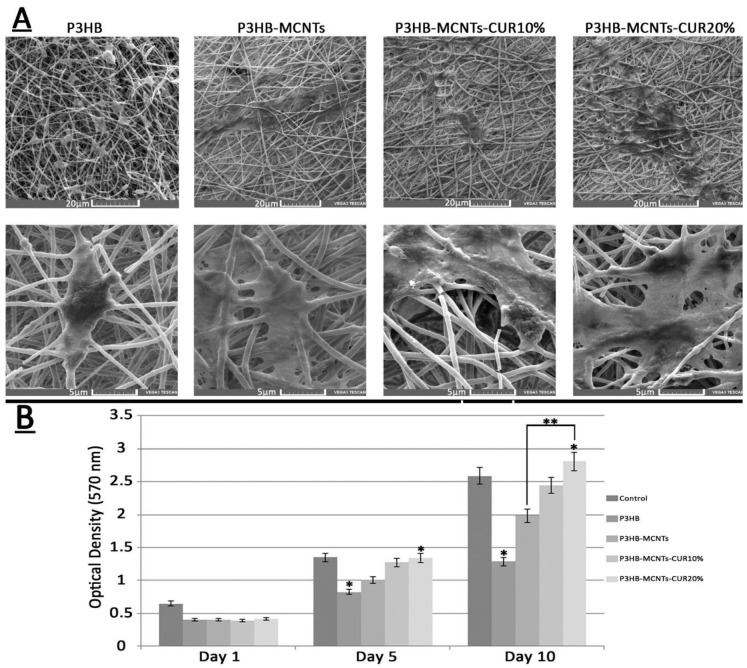
(**A**) MSCs morphology seeded on scaffolds and (**B**) optical density reflecting cell viability, * *p* ≤ 0.05, ** *p* ≤ 0.01.

**Figure 10:**
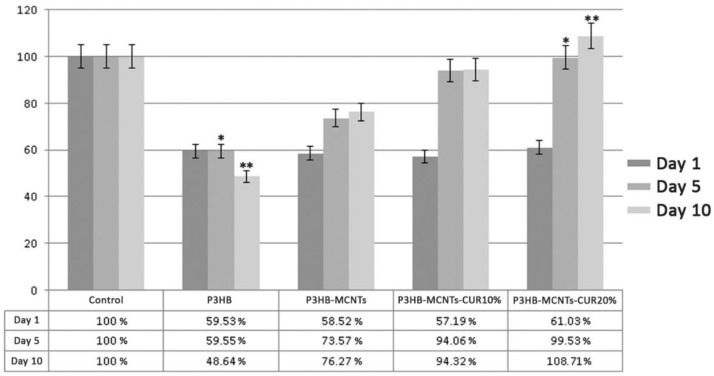
The percentage of cell viability for different groups of scaffolds, * *p* ≤ 0.05, ** *p* ≤ 0.01.

Figure 9 shows SEM pictures of the cultured cells attached onto the scaffolds after ten days (Figure 9A) and the results from MTT assay at the first, fifth, and tenth day of seeding the extracted stem cells onto the scaffolds (Figure 9B). As can be seen from SEM pictures, the stem cells were well attached to the surface of P3HB scaffolds, however the cell attachment for the P3HB-MCNT composites was more apparent and better spread than on scaffolds loaded with CUR. As can be seen from the MTT assay results, a significant increase of cell viability after five days was observed for scaffolds containing MCNTs as well. Our observations confirm that the addition of MCNTs can enhance biological properties of P3HB scaffolds. Figure 10 also shows the percentage of cell viability relative to the control group (scaffold-free). Moreover, increase in cell viability has also been noticed for scaffolds containing 10 wt% CUR, and significantly higher cell viability was observed for scaffolds loaded with 20% CUR (Figure 9). Thus, considering the superior mechanical properties and enhanced biodegradability, along with excellent in vitro cytotoxicity, P3HB-MCNTs-20%CUR material has been selected for further in vivo experiments.

### 3.7. In Vivo Biocompatibility

**Figure 11:**
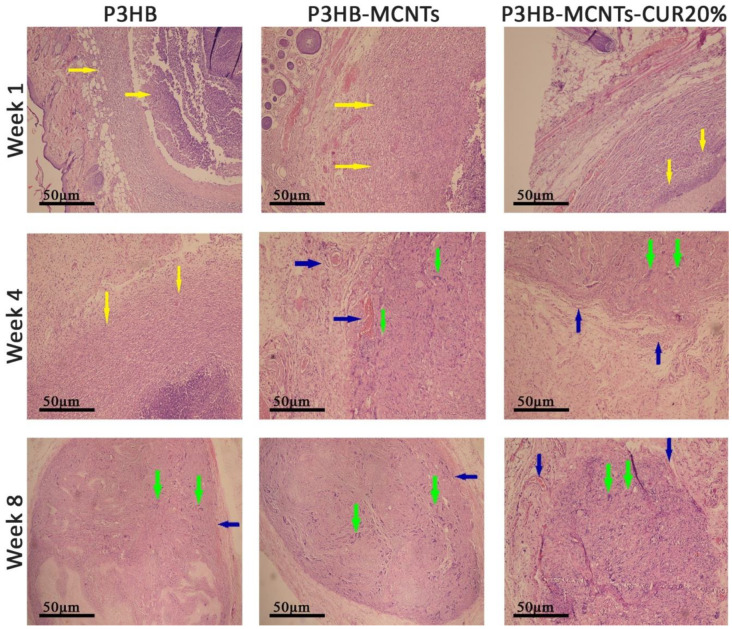
Microscopic images of semithin sections after 1, 4 and 8 weeks of implantation; yellow arrows show inflammation, green arrows show foreign body type giant cell reactions and blue arrows show vessel formation around the scaffolds. Magnification: 400x, H-E staining.

**Figure 12:**
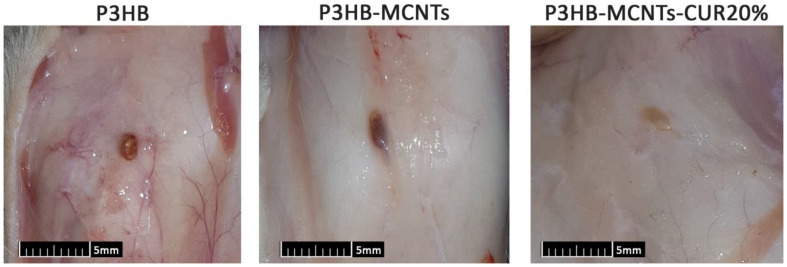
Macroscopic pictures of the remains of scaffolds after 8 weeks of implantation.

Final verification of the expected bioactivity of CUR, specifically in suppression of inflammatory response, was performed by subcutaneous implantation of the material and evaluation of the reaction of the surrounding tissues after a specified time period [51]. In this study, no signs of systemic or neurological toxicity, infection, reduced activity and immobility were observed by monitoring the rats during 8 weeks of implantation. After the first, fourth and eighth weeks of implantation, histological reactions of the surrounding tissues were evaluated by preparing H&E stained histological sections (Figure 11). In group 1 (neat P3HB scaffolds), severe acute inflammatory cell infiltration was observed after one week of intradermal scaffold implantation. This reaction was gradually decreased during subsequent 4 weeks and replaced with chronic inflammatory cells as well as foreign body giant cells. In this way, the scaffold was gradually resorbed. After 8 weeks of implantation, there was evidence of a scaffold remnant. The same reactions occurred for groups 2 (P3HB-MCNTs) and 4 (P3HB-MCNTs-CUR20%), however for group 4, there was no evidence of remaining scaffold material. Notably, there was less acute inflammation in group 4 observed due to the presence of CUR as well as higher resorption of scaffolds. Some vessel formation around the scaffolds can also be seen for groups 2 and 4. Angiogenesis ability of the scaffold material is also crucial in the tissue engineering process. Vessel formation inside engineered tissue structure can mimic a functional blood vessel [52]. Figure 12 shows the macroscopic pictures of the remains of the scaffolds after 8 weeks of implantation; it indicates that the scaffolds have been well degraded, as their original size was 15 mm × 15 mm. These findings confirm that the introduction of CUR into P3HB-MCNTs fibrous scaffolds allows production of biocompatible and biodegradable scaffolds for possible tissue engineering applications.

## 4. Conclusions

New nanocomposite scaffolds containing poly(3-hydroxybutyrate) and multiwalled carbon nanotubes were loaded with curcumin to produce P3HB-MCNTs-CUR nanofibrous materials by electrospinning. Different percentages of curcumin, from 10% to 30%, were added to the P3HB-MCNTs nanocomposite scaffolds to select the best composition for bone tissue engineering applications. Successful incorporation of curcumin into P3HB-MCNT composite was confirmed by infrared spectroscopy and proton NMR, thus indicating the beneficial role of surface modified MCNTs in P3HB scaffold capable of interacting with curcumin. The highest mechanical properties and structural integrity of the fibers was found for scaffolds containing 20 wt% of curcumin. This bioactive compound has also accelerated the hydrolytic degradation in PBS and enhanced hydroxyapatite precipitation form SBF. Moreover, curcumin strongly reduced inflammatory reaction after 8 weeks of in vivo implantation of the scaffolds. Overall, our findings clearly indicate that electrospun scaffolds made of P3HB-MCNTs-CUR20% can be promising structures for tissue engineering applications.

## Figures and Tables

**Table 1:** ^1^H NMR chemical shifts (ppm) of poly(3-hydroxybutyrate)(P3HB), curcumin (CUR), and the P3HB-CUR composite mixture containing MCNTs.

Compound	Moiety	Chemical Shift (ppm)	Integral
CUR	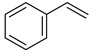	6.472–7.501	12
	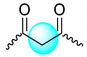	5.810–5.823	2
	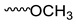	3.967	6
	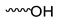	7.585–7.637	2
P3HB	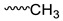	1.278–1.299	3
	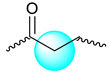	2.449–2.662	2
	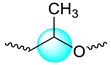	5.220–5.326	1
Mixture	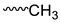	1.283–1.303	3
	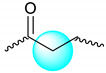	2.195–2.667	2
	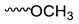	3.973	0.3
	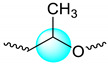	5.248–5.309	1
	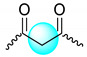	5.825–5.890	0.13
	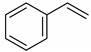	6.476–7.505	1
	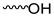	7.589–7.641	0.14
